# Lipids in Equine Airway Inflammation: An Overview of Current Knowledge

**DOI:** 10.3390/ani14121812

**Published:** 2024-06-18

**Authors:** Jenni Mönki, Anna Mykkänen

**Affiliations:** Department of Equine and Small Animal Medicine, Faculty of Veterinary Medicine, University of Helsinki, Viikintie 49, P.O. Box 57, 00014 Helsinki, Finland; jenni.monki@helsinki.fi

**Keywords:** horse, equine, airway inflammation, asthma, lipids, lipidomics, fatty acids, extracellular vesicles

## Abstract

**Simple Summary:**

Horses often suffer from two common respiratory diseases: mild–moderate equine asthma and severe equine asthma. Symptoms of these conditions include coughing, nasal discharge, reduced performance (especially in physically demanding activities), and difficulties breathing. These symptoms mainly result from inflammation deep within the lungs, the severity of which veterinarians and researchers struggle to accurately assess. Even the best method available, obtaining a sample of bronchoalveolar lavage fluid, has limitations. Lipidomics, a relatively unexplored field in veterinary medicine, represents an intriguing avenue for exploration. Lipidomics focuses on studying cellular mechanisms and interactions, which are crucial in inflammation. Researchers aim to uncover fresh insights into equine lower airway diseases using lipidomics, potentially leading to improved treatments. This review article aims to compile the latest knowledge in this area.

**Abstract:**

Mild–moderate and severe equine asthma (MEA and SEA) are prevalent inflammatory airway conditions affecting horses of numerous breeds and disciplines. Despite extensive research, detailed disease pathophysiology and the differences between MEA and SEA are still not completely understood. Bronchoalveolar lavage fluid cytology, broadly used in clinical practice and in equine asthma research, has limited means to represent the inflammatory status in the lower airways. Lipidomics is a field of science that can be utilized in investigating cellular mechanisms and cell-to-cell interactions. Studies in lipidomics have a broad variety of foci, of which fatty acid and lipid mediator profile analyses and global lipidomics have been implemented in veterinary medicine. As many crucial proinflammatory and proresolving mediators are lipids, lipidomic studies offer an interesting yet largely unexplored means to investigate inflammatory reactions in equine airways. The aim of this review article is to collect and summarize the findings of recent lipidomic studies on equine airway inflammation.

## 1. Introduction

Lipids are essential components of all organ systems, serving not only as an energy source and storage form but also contributing to versatile cellular functions [1,2]. Lipids are known to serve as structural components of membranes, act as signalling molecules and circulating hormones (such as oestrogens) [3], provide a platform for membrane protein recruitment, and act as lipid ligands that modulate protein functions [2].

Lipidomics is a field of science that aims to achieve a comprehensive picture of the entire lipidome (i.e., the set of all lipids) in each biological system such as a cell, tissue, body fluid, or organism [4]. Lipidomic techniques were developed in the early 2000s [5] and the number of related scientific reports in the field have sky-rocketed ever since [6]. To date, this subfield of metabolomics has not yet been widely explored in equine medicine [7].

Abnormal tissue lipid profiles and signalling modulate inflammation [8] and the pathogenesis of several diseases, including respiratory diseases in humans [9,10] and horses [11,12,13]. Molecules derived from cellular lipids are active in both proinflammatory and proresolving phases of inflammation [14]. The functions of Specialized Proresolving Mediators (SPMs), such as resolvins, have recently been acknowledged and are subject to intensive research [15].

Lipidomics have been used to investigate mechanisms and treatments of asthma and other lung diseases in humans [9,16,17,18,19,20,21,22], whilst studies in horses have included lipid and/or fatty acid (FA) compositions of plasma, surfactant [11,22], bronchoalveolar lavage fluid (BALF) cells [23], and BALF extracellular vesicles (EVs) [24] from horses affected by different types and severities of airway inflammation. The aim of this review article is to explore the basic concepts of biologically active lipids in the equine body, with special emphasis on the role of lipids in inflammation, and to collect and summarize the findings of recent lipidomic studies on equine airway inflammation.

## 2. Lipids in the Equine Organ System

The digestive system of a horse relies extensively on symbiosis with the microbiome of the caecum and colon [25], where plant fibre indigestible for mammals is fermented by bacteria. The end products of this process, short-chained or volatile fatty acids [26], are absorbed from the hindgut to the horse’s systemic circulation, metabolized in the liver, stored in the fat tissue, and utilized as energy in tissues such as striated muscle [27]. Extra dietary fat is well absorbed from the small intestine and can be used as a part of balanced nutrition plan in horses [27].

In mammals, fat and adipose tissue function not only as energy storage but also an active endocrine organ. Fat tissue contains adipocytes, preadipocytes, fibroblasts, vascular endothelial cells, and all types of immune cells [28]. Adipose tissue secretes adipokines that regulate many vital functions of the body, such as energy metabolism, eating behaviour, peripheral insulin sensitivity, development of the female reproductive system, and inflammation [29].

In both humans and horses, excessive adipose tissue is a potent proinflammatory site [30,31]. When adipocytes expand excessively, they become dysregulated, apoptose, and necrose, and an inflammatory cascade becomes activated. These local inflammatory changes in the tissue can ultimately “spill over” and cause systemic increases in proinflammatory cytokines [31]. In fat tissue, local hypoxia and endoplastic reticulum (ER) stress reduce the release of insulin-sensitizing adipokines, such as adiponectin. Increased levels of cortisol and the activation of proinflammatory cytokine receptors, such as Toll-like receptors (TLRs), further reduce tissue insulin sensitivity. Consequently, the rate of triacylglycerol (TG) synthesis is reduced, the levels of free fatty acids in blood increase, and insulin-mediated glucose uptake decreases in peripheral tissues like muscle [32]. Finally, obesity in both humans and horses often leads to metabolic syndrome [33].

## 3. Structure and Major Cellular Functions of Main Lipid Classes

**Figure 1:**
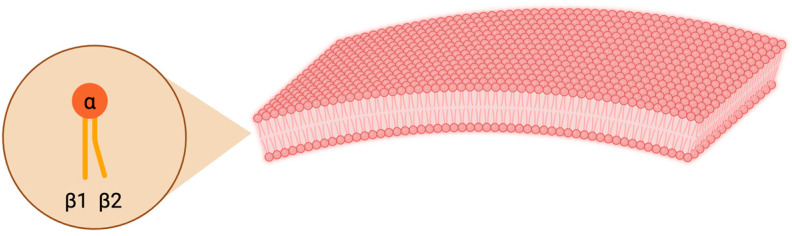
Cellular membrane structure: A phospholipid consists of a hydrophilic head group (α) and hydrophobic tails (β1, β2). Eukaryotic cell membrane bilayers comprise lipids. Created with BioRender.com.

Lipids are typically complex biomolecules, consisting of a hydrophilic head group and hydrophobic tail(s) (Figure 1). FAs are the main building blocks of lipids and the reason for the extreme diversity of lipids [34]. An FA consists of a carboxylic group and a hydrocarbon acyl chain that can vary in both length (number of carbon atoms; C14–C26) and degree of saturation (the number of double bonds between the carbon atoms). FAs containing acyl chains without double bonds are referred to as saturated FAs. FAs containing only one double bond are called monounsaturated (MUFAs) and FAs with multiple double bonds are called polyunsaturated (PUFAs) [35]. The chemical formulas of the FAs are defined by their acyl chain length and by the number of double bonds [36].

The structure of a lipid has direct impacts on its physical properties, biological behaviour, and activity. For example, the double bond in an MUFA or a PUFA is normally in the *cis* configuration, introducing a physical kink in the molecular shape that hinders them from packing closely as the neighbouring chains are pushed away from each other. This causes fats with high PUFA content to remain in a fluid form over a wide range of temperatures [36]. In a membrane bilayer, the three-dimensional form (cylinder-shaped, cone-shaped, or inverted cone-shaped) of its lipids determines the curvature of the membrane [37]. Lipids can be classified as hydrophobic or amphiphilic based on their physical properties. An amphiphilic lipid has properties that permit the formation of cellular membranes (such as a hydrophilic headgroup at one end and a hydrophobic region at the other end) and allow the lipids to line up to form a monolayer. Two of these monolayers then come together to form a membrane, with the water-soluble hydrophilic regions facing the aqueous environment (e.g., extracellular space) and the hydrophobic regions facing each other (Figure 1) [38].

The most common lipids present in biological membranes of mammalian cells can be subdivided into three classes—glycerolipids, sphingolipids, and sterols [35]. Lipids originating from the glycerolipid class that contain phosphate are known as phospholipids, which are the main components of the membranes together with cholesterol (from the sterol class). Table 1 presents the major lipids of the mammalian cell and summarises studies performed mostly on laboratory animals.

## 4. Lipids in Inflammation

All mammals need certain essential FAs to perform vital cellular functions. Linoleic acid (LA; 18:2n-6) is the “parent” omega (Ω)-6 FA, which can be elongated and desaturated in the body to form the long-chain PUFAs dihomo-γ-linolenic acid (DGLA; 20:3n-6) and arachidonic acid (AA; 20:4n-6). In the Ω-3 family, α-linolenic acid (ALA; 18:3n-3) competes for the same elongase and desaturase enzymes to form eicosapentaenoic (EPA; 20:5n-3) and docosahexaenoic (DHA 22:6n-3) acids [26]. Horses can synthesize the longer-chain PUFAs from LA and ALA, which explains the presence of AA, EPA, and DHA in their cell membranes, even in the absence of these FAs in their diet [55]. The Ω-3 and Ω-6 FAs compete for available enzymes and opportunities to be incorporated into cell-membrane phospholipids. This competition occurs in a dose-dependent fashion. For example, when a greater amount of EPA is present, it will be incorporated into cell membranes, partially at the expense of AA [8,56]. The resulting changes in membrane fluidity and integrity, cell receptor signalling, and protein synthesis can alter the body’s response to trauma and infection. When EPA concentration in the membrane is higher, less substrate is available for the synthesis of eicosanoids (AA derivates) during the inflammatory process [8].

The acute phase of inflammation in the body is a complex series of crossing chain reactions between different cells and cytokines, forming a vital defence mechanism [38]. However, returning to homeostasis is also needed after acute insult, otherwise an unwanted state of chronic inflammation prevails [15]. Inflammation does not automatically cease without mechanisms of resolution, which encompass active dampening of inflammation and systematic correction of the damage caused by the inflammatory process. SPMs are lipid mediators that are part of a larger family of proresolving molecules [14]. The production of SPMs is not constitutive and is only initiated after an acute inflammatory response [57]. SPMs have many active roles in returning tissue homeostasis, such as reducing leukocyte recruitment, inducing neutrophil apoptosis, and enhancing efferocytosis at the site of inflammation [58].

Due to their functional potential in pico–nanomolar concentrations, actions as hormone-like autocrine or paracrine molecules, and rapid metabolism rate, detection of SPMs is rather difficult. Their presence was only discovered in the late 1990s and, thus, they have been far less studied than mediators of proinflammatory pathways [59]. Some specific actions of SPMs on immune cell function have been discovered, however. For example, lipoxins boost monocyte chemotaxis and adhesion. Maresins enhance the polarization of macrophages towards an anti-inflammatory phenotype and improve their phagocytosis and efferocytosis activities, eventually leading to the generation of proresolving macrophages [57]. In adaptive immunity, D-series resolvins diminish the differentiation of T cells to proinflammatory helper T (Th) cells and reduce cytokine production of activated Th1, Th17, and CD8+ cytotoxic T cells [60].

**Figure 2:**
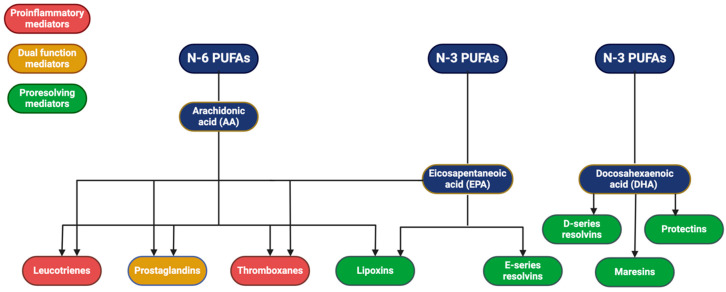
Lipid mediator pathways. Created with BioRender.com.

As mentioned, the specific anti-inflammatory actions of DHA and EPA are still somewhat unclear. It is known, however, that Ω-3 FAs are extremely important in the process of spontaneous resolution of acute inflammation. They are needed as precursors for the biosynthesis of both anti-inflammatory and proresolving lipid mediators that are enzymatically biosynthesized from PUFAs (Figure 2). The circulating DHA and EPA dissolve in inflammatory exudate, such as oedema fluid, to reach the site of action and act as a source for resolvins at the site of infection or tissue trauma [61].

The body’s failure to resolve an inflammatory reaction can lead to multiple chronic pathologies [61]. Low levels of circulating SPMs have been reported in certain lung diseases in humans, such as severe asthma and cystic fibrosis [62]. Interestingly, the administration of SPMs can attenuate disease symptoms, which has also been shown in vivo [63]. Thus, SPMs offer a potential explanation for n-3 PUFA-related benefits in inflammatory conditions. The latest advancements in lipidomics research methods continue to aid the investigation of the direct effects of n-3 PUFA supplements on SPM concentrations in various tissues [14]. To date, SPMs remain largely unstudied in horses [58].

## 5. Lipidome Studies in Equine Health and Disease

Lipids are more difficult to analyse compared to many other biomolecules due to their considerable diversity and insolubility in water. Early studies in lipidomics employed chromatographic methods, such as partition chromatography (TLC) and gas chromatography (GC), which can only distinguish lipid classes. The development of mass spectrometry (MS) and its many applications have made a more comprehensive analysis of the lipidome possible [4]. Various equine sample types can be analysed using different lipidomics analysis methods, such as FA analysis using gas chromatography, global lipidomics using MS, lipid mediator (LM) analysis using targeted MS, and lipid class analysis by high-performance, thin-layer chromatography.

Chloroform-containing liquid–liquid extraction methods, such as the Bligh–Dyer or Folch extraction protocol [64], are most often used to prepare a sample. If the sample is not in fluid form, it is first homogenized and dissolved into a mixture of chloroform and methanol. An antioxidant is added to prevent oxidation of double bonds. A lipid standard mix is then added to correct later differences in lipid extraction efficiency and ionization. Lipids are recovered in the organic phase after centrifugation and evaporation. Alternatively, the raw extract is applied to a resin in a column for solid-phase extraction and elution. After these steps, lipid extracts are finally subjected to MS analysis [4]. Although the lipidome has been investigated for many equine organ systems and diseases, utilizing various lipidomic techniques, there are only a few reports from each subfield. Some clinical lipidome studies in horses are introduced in Table 2. Most of the studies employed untargeted/shotgun lipidomics using some form of MS.

## 6. Lipids in Respiratory Diseases

In human medicine, the mechanisms of many lower airway diseases have been explored utilizing lipidomics [75]. For instance, studies on human asthma [21,75], cystic fibrosis [18,76], bacterial pneumonia [17], lung injury caused by smoke inhalation [19], and COVID-19 [20,77] have revealed altered lipid profiles in the lungs of affected patients. Here, we focus on lipidomic studies of asthma in humans and horses. The clinical presentation of asthma as well as some immunological features are similar in both species, but differences are also present and should be kept in mind when comparing studies between the two species [78].

Increasing evidence suggests abnormal lipid metabolism in asthmatic people [79,80]. However, it is important to acknowledge that lipidomic studies may explore either FA, lipid, or lipid mediator (LM) profiles and use various sample types, making comparisons between studies on human asthma challenging. Furthermore, the lipid composition and metabolism of horses differs from that of humans [81].

Differences in lipid composition between human asthma patients and controls have been found in plasma [82,83]; bronchoalveolar cells [84]; alveolar macrophages, bronchial epithelial cells, and alveolar type II cells [85]; EVs in BALF [10]; and sputum [86]. Exhaled breath condensate demonstrated no differences between cases and controls, which may be due to very diluted concentrations of lipids in the sample [87]. Research on lipidomics in equine airway inflammation is still in its infancy.

Similarly to humans, current lipidomic studies in equine medicine have diverse areas of focus, such as FA, lipid, or LM profiles, or combinations thereof. In horses, plasma and cell-free surfactant samples have been the most common sample types investigated [11,22,88]. The BALF cell lipidome [23] and BALF EV FA profiles [24] have recently been investigated in healthy horses and during different stages of airway inflammation.

Wang et al. [83] found some potential lipid biomarkers of human asthma in plasma samples analysed by high-performance liquid chromatography with quadrupole time-of-flight mass spectrometry (HPLC-QTOF-MS). They investigated glycerophospholipid (GP) metabolites and found that phosphatidic acid (PA) and phosphatidylglycerol (PG) levels were higher in asthma patients’ plasma. In addition, plasma PA, PG, and PE levels were higher in patients with eosinophilic asthma than non-eosinophilic asthma. Another study [82] also found differences in the plasma lipid species of mild and moderate asthma cases compared to controls using liquid chromatography (LC)-MS/MS. This study found that PE (38:1) might be a useful biomarker for human asthma, as it effectively discriminated the asthma cases from the controls. The plasma concentration of PE (18:1p/22:6), PE (20:0/18:1), PE (38:1), SM (d18:1/18:1), and TG (16:0/16:0/18:1) increased with asthma severity while PI (16:0/20:4), TG (17:0/18:1/18:1), phosphatidylglycerol (PG) (44:0), Cer (d16:0/27:2), and lysophosphatidylcholine (LPC) (22:4) decreased with asthma severity [82]. Kang et al. [84] used HPLC-QTOF-MS to reveal the lipid profiles of BALF samples from people with severe bronchial asthma (SBA) that either received steroid treatment or not, and healthy controls. The individual quantity levels of the six classes of lipids were higher in asthmatics. In the SBA subjects, the PC, PG, PS, SM, and TG levels were similar to the levels observed in the control group. Using differentially expressed lipid species, 34 lipid biomarker candidates were identified with high-prediction performance between asthmatics and controls, revealing specific characteristics of lipid phenotypes in asthmatic patients.

### 6.1. Sphingolipid (SM, Cer, and HexCer) Species

Cer and other sphingolipids (SM and HexCer) have some lung-specific functions and several studies have identified Cer as a potential lipid biomarker of human asthma [50,89]. Sphingolipids are minor structural components of surfactant, also regulating their synthesis and release. They have crucial roles in endothelial barrier maintenance [9] and EV biogenesis [90]. In the lung, sphingolipid synthesis and ceramide levels are vital for processes such as epithelial apoptosis, endothelial permeability and immune cells mobilization, mucus production, and pathogen clearance [9]. Increased amounts of Cer were detected in the airway epithelium cells of guinea pigs in an allergic asthma model [91]. Zehethofer et al. [16] also detected increased levels of Cer in mouse and human bronchus tissue samples in response to allergen challenge.

The roles of sphingolipids in equine airway inflammation have been minimally investigated. In a study by Christmann et al. [22], proportions of Cer 18:1;O2/16:0, Cer 18:1;O2/24:0, Cer 18:0;O2/16:0, and hydroxy ceramide (OH-Cer) 18:1;O2/24:0 were elevated in crude surfactant pellets of clinical SEA cases of various horse breeds (four American Quarter Horses, one Thoroughbred, one Morgan, one Paint Horse, one Warmblood, one Tennessee Walking Horse, one Appaloosa, one Arab, and one mixed-breed). In a recent study by our research group [23], Cer and HexCer lipid classes in BALF cell pellets decreased together with BALF cell counts and neutrophil percentages after subclinical airway inflammation was induced with a bedding challenge. This might indicate that both Cer and HexCer contribute to inflammation resolution in horse lungs. Contrary to the study of Christmann et al. [22] on surfactant, Cer 18:1;O2/16:0 was lower and HexCer 18:1;O2/24:0 higher in the BALF cells after the bedding challenge in healthy horses [23]. Cer 18:1;O2/24:0 was consistently higher consistently in both surfactant [22] and BALF cells [23]. The differences between these results are most likely due to two factors, namely the degree of inflammation (asthmatic horses vs. subclinical inflammation in healthy horses) and the sample type, since Cer is present in surfactant in small amounts although they are important cellular membrane elements. While Cer has been reported to be highly expressed in asthmatics [9,16,91], reduced amounts of ceramide phosphates and Cer were measured in the EVs of horses with asthma [10].

### 6.2. Phosphatidylinositol (PI) Species

In human medicine, PI has been identified as an indicator of lung immaturity and injury. However, PI is not only solely proinflammatory but also seems to have anti-inflammatory properties. In a study by Numata et al. [45], PI inhibited human respiratory syncytial virus (RSV) infections in laboratory animals. The roles of PI in equine lungs have not been studied extensively. Term foals of Quarter Horses and various light breeds [92] had higher concentrations of PI in their surfactant compared with adult horses. In other species, young individuals have higher serum concentrations of the carbohydrate myoinositol, which promotes maturation of surfactant and acts as a precursor for PI. In human neonates, a decrease in plasma myoinositol concentration is associated with a higher risk of acute respiratory distress syndrome (ARDS) [93]. In a recent equine study, Bazzano et al. [13] analysed BALF and exhaled breath condensate with proton nuclear magnetic resonance (1H-NMR), showing that EA horses had lower levels of myoinositol in BALF compared to controls.

In our study on equine BALF cells [23], we found an increased mol% of PI 38:4 in the BALF cells from horses that were housed on wood shavings compared to peat bedding. This finding can be explained by the signalling role of arachidonic acid (20:4n-6) that is specifically released by PI 38:4 for the production of lipid mediators such as leukotrienes and prostaglandins (e.g., LTB4 and PGE2) [40,94,95]. Leukotrienes, including LTB4, contribute to the pathogenesis of human asthma [96,97]. This finding, together with increased BALF cell counts suggests a proinflammatory state induced by housing the horses on wood shavings. Interestingly, in the same study, four additional polyunsaturated PI species were among the top 25 lipid species that increased in BALF cells [23].

### 6.3. Phosphatidylcholine (PC) Species and Surfactant

Surfactant contains lipids from several classes, including phospholipids, TG, and cholesterol, with PC covering approximately 80% of the lipids in surfactant [41]; PC 32:0 is the major phospholipid species of surfactant [98]. Christmann et al. [92] also found this to be true in horses, as PC contained 90% of the surfactant phospholipids in adult individuals of various light-build horse breeds. Pulmonary surfactant is essential for life due to its actions in the lung, where it maintains the surface tension that prevents the alveoli from collapsing [99]. Alveolar type II epithelial cells synthesize surfactant in their endoplasmic reticulum (ER). From ER, surfactant is transported and stored in intracellular storage organelles called lamellar bodies. Lung surfactant is secreted via exocytosis from these cells. Secreted extracellular surfactant removes foreign material and sloughed cells from the airways [99]. Pulmonary alveolar macrophages (PAMs) reside at the alveolar surface, as do alveolar type II epithelial cells; 10–15% of the surfactant secreted by the alveolar type II cells is degraded by the PAMs [100]. Altered surfactant clearance by the PAMs has been implicated as a reason for the surfactant alterations associated with acute respiratory distress syndrome (ARDS). A study by Forbes et al. [101] demonstrated that depletion of the PAMs caused a several-fold increase in surfactant pool sizes, which suggests that PAMs are essential for surfactant homeostasis in a healthy lung of rats. PAMs may also contribute to the regulation of the amount and composition of surfactant lipids, e.g., by employing ABC cassette transporters [102,103].

In human asthma, surfactant dysfunction, changes in surfactant phospholipid composition, and alterations in surfactant distribution are parts of the disease mechanism [99,104]. Comparatively, decreased amounts of surfactant were found in horses with SEA, and its lipid composition was changed compared with healthy controls [105]. Christmann et al. [11] investigated surfactant samples from healthy and asthmatic horses exposed to the same environmental conditions. They detected increased amounts of cyclic phosphatidic acid (cPA) and DAG in surfactant from severely asthmatic horses during exposure to hay. Later, the same group [22] performed lipidomic analysis of surfactant and plasma from horses affected by different forms of asthma and healthy controls. Horses with SEA had altered phospholipid content and composition of surfactant, whereas only mild changes were observed in horses with mild neutrophilic equine asthma. The plasma lipidomic profile was significantly different between groups of asthmatic horses and healthy controls.

Albornoz et al. [12] performed a metabolomics analysis on BALF samples retrieved from asthmatic horses and healthy horses during an episode of experimentally induced (lipopolysaccharide inhalation) airway inflammation. From both groups, BALF supernatant samples were subjected to metabolic analysis with GC-MS. Decreases in palmitic (16:0), palmitoleic (16:1n-7), and oleic acids (18:1n-9) were found in both groups. This finding could be related to a change in the composition of pulmonary surfactant, as these FAs are the main components of PC.

In our recent study investigating equine BALF cells [23], some of the most interesting results were the increases in lipid species that contain 16:0 acyl chain (such as PC 32:0), which were apparent together with lower BALF cell count and neutrophil percentage after horses returned from wood shavings to peat bedding, indicating resolution of airway inflammation. Thus, it appears that phospholipid PC 32:0 is also a feature of healthy airway cells in horses. Phospholipids have been suggested to be largely responsible for establishing the hydrophobic surfaces of mucous membranes, providing barriers for the underlying tissues. Exogenous PC has shown promise as an anti-inflammatory agent in animal models of gastrointestinal inflammation [106]. Furthermore, the proportions of two PC species PC 32:0 (main molecular species 16:0_16:0) and PC 36:4 (containing also 20:4n-6, arachidonic acid) showed a strong negative correlation, making their ratio a promising marker of inflammation versus resolution, which was already detected in this study at the subclinical stage.

## 7. Extracellular Vesicle Lipidome in Human and Equine Asthma

EVs are cell-derived structures with a double lipid membrane that mediate cell-to-cell signalling. In humans, both the amount of EVs increases and their FA composition changes in human asthma [10]. The proportion of phosphatidylglycerol, ceramide phosphates, and Cer reduces while SM 34:1 increases in EVs of asthmatics exposed to smoke compared with healthy controls [10]. In asthmatic horses, the number of EVs did not change. However, the proportions of palmitic acid (16:0) decreased and those of EPA and DGLA increased in EVs of horses with SEA [24]. Therefore, EVs may serve as a targeted delivery system and carry FAs that can modulate and ameliorate inflammation in asthma.

## 8. Modulating the Airways’ Inflammatory Reactions from a Lipidomics Perspective: Role of Diet and FA Supplements

In Section 3, the role of lipids—omega FAs specifically—in inflammation were summarized. Forage is the main component of a natural equine diet and forage-based, good-quality diets likely meet the essential FA requirements of the animal [26]. While pasture grass has a very low fat content, the fat it contains is rich in Ω-3 PUFAs [107]. Horse muscle tissue has been shown to contain a higher amount of LA when horses have been grazing instead of being fed high-concentrate feed [108]. However, many horses may not either permanently or seasonally have access to pasture. In these cases, the type of forage and possible nutritional supplements become of interest, especially in horses with chronic respiratory conditions. Although horses are natural herbivores, marine-based Ω supplements can be incorporated into their diets [109]. Marine-based Ω-products have shown superiority to plant-based products in most studies that have evaluated their anti-inflammatory/proresolving effects in horses [109,110,111,112,113,114].

In horses, haylage feeding causes greater resolution of airway inflammation than other low-dust forage products (pelleted hay and steamed hay) [58,115]. In one study, haylage increased the ratio of anti-inflammatory to proinflammatory FAs in the horse’s plasma, where the EPA:AA ratios were higher in horses after eating haylage for 6 weeks compared with baseline or horses eating steamed or dry hay [58]. However, forage had no effect on the plasma levels of LA, γ-linolenic acid, ALA, steridonic acid (SDA), eicosadienoic acid, DGLA, AA, docosadienoic acid, DHA, or EPA. Furthermore, no effect of time, forage, or forage over time was observed on plasma concentrations of SPMs resolvin D1 (RvD1) or resolvin E1 (RvE1).

The effects of PUFA supplements on equine airway inflammation have been studied. LPS-stimulated BALF cells from horses fed corn oil showed a higher production of proinflammatory eicosanoid PGE2 than those from horses fed fish oil. Both oil diets increased BALF cell TNF-α production and phagocytic activity. Khol-Parisini et al. [116] showed that when horses were fed sunflower oil rich in ALA or seal blubber oil rich in DHA, DLA, and EPA, the supplementary FAs were incorporated into leukocyte cell membranes. Compared to sunflower oil, the Ω-6:Ω-3 FA ratios in plasma, leukocyte phospholipids, and BALF leukocyte counts were lower after seal blubber oil supplementation. However, neither pulmonary function nor clinical signs of SEA were markedly affected by the different dietary supplements. Nogradi et al. [117] showed that feeding asthmatic horses (MEA and SEA) an algae supplement containing Ω-3 (1.5–3 g DHA/d) for two months provided an additional benefit to a low-dust diet on airway inflammation. Increased plasma DHA levels, peaking at 4 weeks, were observed together with improvements in clinical signs, lung function, and BALF neutrophil percentage when compared with the placebo group treated only with a change of diet. In this study, a larger dose (60 g/d) of the PUFA supplement offered no greater benefits than the recommended dose (30 g/d).

## 9. Conclusions and Future Directions

Lipidomics is a sensitive tool to detect nuances in different stages of equine lower airway inflammation. Current knowledge in the field remains limited. In the future, lipidomic techniques may help to differentiate endotypes and reveal pathomechanisms of EA. Lipidomics’ relation to inflammation also presents wider opportunities in translational asthma research between humans and horses. Therapies based on modulating pathological inflammatory reactions in asthmatic horses utilizing proresolving pathways seem promising.

## Figures and Tables

**Table 1:** Examples of functions of certain lipids in the cells.

Lipid Class	Location and Main Functions
Phosphatidylcholine (PC) and phosphatidylethanolamine (PE)	Most abundant structural phospholipids of the membranes in mammalian cells [39]. Major storage for arachidonic acid (AA), among other signalling lipid mediators [40]. AA can be released from these lipids by the action of phospholipase A2 (PLA2) enzymes [40]. PC is also the main component of lung surfactant [41]. PC:PE ratio in the endoplasmic reticulum (ER) membranes is important; a reduction in PC:PE leads to loss of membrane integrity due to bilayer stress caused by the differences in the biophysical properties of PC and PE [42]. PE is most concentrated in the membranes of ER and mitochondria. PE is involved in membrane structure, curvature, and dynamics. It promotes membrane budding and vesicle fission and fusion [40].
Phosphatidylserine (PS)	Membrane phospholipids in mammalian cells with multiple roles in cell signalling, endocytosis, platelet activation, blood coagulation, and apoptosis [43]. In healthy cells, PSs are confined to the inner bilayer of the plasma membrane, whereas apoptotic cells translocate PSs to the outer leaflet of the plasma membrane [35]. Exposure of PSs to the outer leaflet is a critical signal to macrophages to remove the damaged cells [44]. PSs activate kinases and many other membrane proteins and promote cell fusions of immunologically active cells [43].
Phosphatidylinositol (PI)	Membrane phospholipids in mammalian cells with numerous roles in cell signalling [45]. PI’s major function is to act as a substrate for making phosphorylated derivatives and PLA2 [35]. PI’s headgroup is remarkably versatile: three of the five hydroxyls can be phosphorylated, resulting in seven different phosphoinositides [43], which, in turn, can recruit and activate cytosolic proteins. Many proteins contain lipid-binding domains that can recognize a specific phosphoinositide. PIs also form protein anchors in membranes [46].
Sphingomyelin (SM)	Membrane phospholipids of mammalian cells; consist of a ceramide backbone bound to a phosphorylcholine molecule [35]. SMs are vital in viscous microdomain formation (so-called lipid rafts) in the cell membrane [47,48] and serve as precursors for sphingolipids in the sphingomyelin cycle [48].
Ceramide (Cer)	Sphingolipids found in mammalian cell membranes in small but varying amounts. Cers are the precursors for SMs [35]. Despite their minimal presence, they are key mediators of apoptosis, autophagy, selective degradation of mitochondria, cell-cycle arrest, and natural aging [49]. Cers are involved in numerous pathological states of the body, such as inflammation [9,50]. Cell stressors, such as ionizing radiation and chemotherapeutic drugs, can trigger Cer accumulation [35].
Hexosylceramide (HexCer)	Minor components of mammalian cell membranes; belong to the group of cerebrosides within the sphingolipids [35]. HexCers participate in forming microdomains (rafts) and influence membrane stability and function, especially in the nervous system [51]. Regulators of endothelial permeability, functioning, for example, in preventing trans-epidermal water loss [48].
Cholesterol esters (CE)	Belong to the group of cholesterol lipids within the sterol lipids [35]. Storage lipids in cytosolic lipid droplets and circulating lipoprotein particles. CE content varies depending on nutritional and metabolic conditions [52]. CEs transport cholesterol in plasma and in cells in lipid droplets and buffer excess cholesterol [52].
Triacylglyserol/triglyceride (TG)	Storage lipids in cytosolic lipid droplets found especially in adipocytes and in circulating lipoprotein particles [53]. TG acyl chains can produce membrane and signalling lipids [54]. TG content varies depending on the animal’s nutritional and metabolic status [53].

**Table 2:** Examples of equine lipidome studies.

Reference	Sample Type	Horses	The Main Findings Summarized
Wood et al. (2016) [65]	sperm, seminal plasma	6 healthy stallions	Individual ether glycerophospholipids and seminolipids essential for membrane lipid raft function in equine sperm. Equine sperm contain OAHFAs. Potential roles of diacylglycerols as both important structural and signalling molecules in sperm are presented.
Wood et al. (2018) [66]	amniotic fluid	6 pregnant mares	Detection of very-long-chain dicarboxylic acids, cholesterol sulphate, and OAHFAs in amniotic fluid of normal pregnancies in mares.
Barreto et al. (2020) [67]	colostrum	34 lactating mares	Variation in the milk’s lipid composition according to the lactation stage.
Yuan et al. (2017) [68]	urine	1 healthy mare	Five TGs, two phosphatidic acids, two PEs, are two phosphatidylethanols identified in mare’s urine.
Kosinska et al. (2021) [69]	synovial fluid	15 humans, 13 horses	Horse synovial fluid contained about half of the PL content of human synovial fluid; changes in PL composition are characteristic for each species.
Elzinga et al. (2016) [70]	plasma	14 cases, 9 controls	EMS horses had increased plasma TG, diacylglycerides, monoacylglycerides, and Cer. Plasma SM, sulfatide, and choline ether lipids were lower in EMS horses.
Coleman et al. (2019) [71]	serum	20 cases, 20 controls	Lipid profile in obese horses closely resembled that of obese humans, humans with metabolic syndrome, or both. For example, CE, diacylglycerol (DAG), and PC classes were increased in obese horses.
Wood et al. (2018) [72]	serum	15 unvaccinated, naturally infected; 15 vaccinated; 15 unvaccinated and unexposed	CPAs, diacylglycerols, and hydroperoxide oxidation products of choline plasmalogens are increased in the serum of naturally *Leptospira*-infected and *Leptospira*-vaccinated horses. TGs were only elevated in the serum of infected horses. SMs were increased only in the serum of vaccinated horses.
Sanclemente et al. (2022) [73]	plasma	9 cases, 4 controls	Ageing and *Rhodococcus equi*-infection-induced changes in the plasma lipidome of foals.
Hallamaa and Batchu (2016) [74]	serum	10 cases, 10 controls	IBH horses had lower total concentrations of PC and SM.

OAHFA = (O-acyl)-ω-hydroxy-fatty acids; PL = phospholipid; TG = triacylglycerol; PE = phosphatidylethanolamine; EMS = equine metabolic syndrome; CPA = cyclic phosphatidic acid; SM = sphingomyelin; IBH = insect bite hypersensitivity; PC = phosphatidylcholine.

## Data Availability

Data sharing is not applicable.
